# Supplementary material for: Consumption of Wild Rice (Zizania latifolia) Prevents Metabolic Associated Fatty Liver Disease through the Modulation of the Gut Microbiota in Mice Model
Source: Int J Mol Sci. 2020 Jul 29;21(15):5375. doi: 10.3390/ijms21155375 (PMC7432455; doi:10.3390/ijms21155375)
Supplement: Supplementary file 1 [file ijms-21-05375-s001.zip › Tables S1-3.docx]

**Supplementary Materials (Tabels 1–3 out of 4. Table S4 is in a separate Excel file)**

**Table S1.** Composition of experimental diets.

| \| **Ingredients** \| \| --- \| | Normal Chow Diet | High Fat Diet | Low Dose Wild Rice Diet | High Dose Wild Rice Diet |
| --- | --- | --- | --- | --- | --- |
| Casein, 80 Mesh | 230 | 215 | 215 | 215 |
| Sucrose | 310 | 265 | 0 | 0 |
| Corn Starch | 295 | 258 | 0 | 0 |
| Cellulose, BW200 | 50 | 50 | 50 | 50 |
| Lard | 0 | 100 | 100 | 100 |
| Soybean Oil | 70 | 0 | 0 | 0 |
| Custard Powder | 0 | 50 | 50 | 50 |
| Cholesterol | 0 | 15 | 15 | 15 |
| Choline Bitartrate | 0 | 2 | 2 | 2 |
| Vitamin Mix | 10 | 10 | 10 | 10 |
| Mineral Mix | 30 | 30 | 30 | 30 |
| DL-methionine | 3 | 3 | 3 | 3 |
| Choline Chloride | 2 | 2 | 2 | 2 |
| Wild rice | 0 | 0 | 261.5 | 523 |
| Total | 1000 | 1000 | 1000 | 1000 |

**Table S2.** List of primers used in this study.

|  | **Sequences (5’-3’)*** | **Product Size (bp)** | **Annealing Temperature(°C)** | **References** |
| --- | --- | --- | --- | --- |
| **Bacterial 16S rRNA gene** | | | | |
| V3region | F:NNNNNNNACTCCTACGGGRSGCAGCAG （F341） | About 200 | 65–55 | Masella et al. ,2012 |
|  | R: GGACTACVVGGGTATCTAATC （R806） |  | (touch down) |  |
| **Mouse gene** | | | | |
| β-actin | F：CCTAGACTTCGAGCAAGAGA | 140 | 140 | Kazuyuk et al. ,2015 |
|  | R：GGAAGGAAGGCTGGAAGA |  |  |  |
| IκB-α | F：ATGGAAGTGATTGGTCAGGTGA | 184 | 184 | Kazuyuk et al. ,2005 |
|  | R：AGGCAAGATGGAGAGGGGTATT |  |  |  |
| NFκB-p65 | F：TCTGTTTCCCCTCATCTTT | 165 | 165 | Bagul et al. ,2015 |
|  | R：TGGTATCTGTGCTTCTCTC |  |  |  |

* The NNNNNNNN was the unique 8-base barcode which was used to sort PCR products into different samples.

References

1. Iwai, Kazuyuki, et al. "IkB-α-specific transcript regulation by the C-terminal end of c-Rel. *FEBS Lett.* 579.1 (2005): 141-144.
2. Bagul, Pankaj K., et al. "Resveratrol ameliorates cardiac oxidative stress in diabetes through deacetylation of NFkB-p65 and histone 3."*The Journal of nutritional biochemistry*26.11 (2015): 1298-1307.
3. Andre P Masella, Andrea K Bartram, Jakub M Truszkowski, Daniel G Brown and Josh D Neufeld. PANDAseq: paired-end assembler for illumina sequences. BMC Bioinformatics 2012, 13:31.

**Table S3.** Sequencing data statistics of samples in this study.

| **Sample ID** | **PE Reads** | **Clean Tags** | **AvgLen (bp)** | **Q20 (%)** | **Q30 (%)** | **GC (%)** | **Effective (%)** | **OTUs** |
| --- | --- | --- | --- | --- | --- | --- | --- | --- |
| NCD1 | 60501 | 54427 | 417 | 0.953 | 0.9196 | 0.5326 | 89.96049652 | 637 |
| NCD2 | 62916 | 54645 | 412 | 0.958 | 0.9265 | 0.5358 | 86.85390044 | 648 |
| NCD3 | 57175 | 48489 | 417 | 0.96 | 0.9292 | 0.5287 | 84.80804547 | 611 |
| NCD4 | 61565 | 53400 | 416 | 0.956 | 0.9232 | 0.5319 | 86.73759441 | 599 |
| NCD5 | 58515 | 48059 | 418 | 0.96 | 0.9296 | 0.5275 | 82.1310775 | 601 |
| NCD6 | 58552 | 49573 | 418 | 0.963 | 0.9349 | 0.528 | 84.66491324 | 583 |
| NCD7 | 63272 | 56537 | 417 | 0.961 | 0.9318 | 0.5314 | 89.3554811 | 642 |
| NCD8 | 58657 | 47183 | 415 | 0.96 | 0.9297 | 0.5386 | 80.43882231 | 640 |
| HFD1 | 57206 | 45230 | 418 | 0.964 | 0.9368 | 0.522 | 79.06513303 | 692 |
| HFD2 | 56232 | 45199 | 415 | 0.962 | 0.9332 | 0.5305 | 80.37949922 | 636 |
| HFD3 | 55677 | 46657 | 420 | 0.961 | 0.9311 | 0.5279 | 83.79941448 | 581 |
| HFD4 | 57833 | 47111 | 418 | 0.962 | 0.9328 | 0.5308 | 81.46041188 | 553 |
| HFD5 | 58844 | 48166 | 410 | 0.966 | 0.9395 | 0.534 | 81.85371491 | 656 |
| HFD6 | 60581 | 50337 | 416 | 0.963 | 0.935 | 0.5288 | 83.09040788 | 688 |
| HFD7 | 55674 | 44608 | 414 | 0.962 | 0.9326 | 0.5362 | 80.12357653 | 698 |
| HFD8 | 55406 | 49685 | 417 | 0.962 | 0.9335 | 0.526 | 89.67440349 | 563 |
| HWR1 | 61522 | 53922 | 417 | 0.951 | 0.9164 | 0.5309 | 87.64669549 | 560 |
| HWR2 | 61879 | 52823 | 417 | 0.959 | 0.9279 | 0.5288 | 85.36498651 | 566 |
| HWR3 | 64012 | 55409 | 419 | 0.961 | 0.9319 | 0.5309 | 86.56033244 | 569 |
| HWR4 | 49505 | 41401 | 418 | 0.945 | 0.9069 | 0.5316 | 83.62993637 | 546 |
| HWR5 | 61510 | 50496 | 417 | 0.957 | 0.9252 | 0.5273 | 82.09396846 | 596 |
| HWR6 | 63577 | 53021 | 417 | 0.96 | 0.9306 | 0.5292 | 83.39651132 | 619 |
| HWR7 | 56852 | 50166 | 421 | 0.948 | 0.9122 | 0.5234 | 88.23963977 | 511 |
| HWR8 | 50113 | 42212 | 419 | 0.96 | 0.9297 | 0.5306 | 84.23363199 | 606 |
| Average | 58649 | 49531.5 | 416.7916667 | 0.959 | 0.9283 | 0.5301 | 84.39844145 | 608.375 |
| Totle | 1407576 | 1188756 | 10003 | 23.01 | 22.28 | 12.723 | 2025.562595 | 14601 |
